# Effect of an herbal capsule on chronic constipation among menopausal women: A randomized controlled clinical trial

**DOI:** 10.22038/AJP.2019.13109

**Published:** 2019

**Authors:** Paria Eliasvandi, Laleh Khodaie, Sakineh Mohammad Alizadeh Charandabi, Mojgan Mirghafourvand

**Affiliations:** 1 *Department of Midwifery, School of Nursing and Midwifery, Tabriz University of Medical Sciences, Tabriz, Iran*; 2 *Department of * *Traditional Medicine* *, Faculty of * *Traditional Medicine* *, Tabriz University of Medical Sciences, Tabriz, Iran*; 3 *Department of Midwifery, Faculty of Nursing and Midwifery, Tabriz University of Medical Sciences, Tabriz, Iran*; 4 *Department of Midwifery, Social Determinants of Health Research Center, Tabriz University of Medical Sciences, Tabriz, Iran*

**Keywords:** Herbal capsule, Chronic constipation, Menopause

## Abstract

**Objective:**

Chronic constipation is frequently observed in postmenopausal women. An herbal combination including clover plants, Roman anis or Anisone, green anis or fennel, green raisins, Alhagi maurorum, violets, Terminalia chebula, senna and golqand has been introduced in traditional books as an effective laxative. Thus, the present study aimed to assess the effectiveness of the combined herbal capsule on chronic constipation in postmenopausal women.

**Materials and Methods:**

This triple blinded, randomized placebo-controlled trial was conducted on 64 postmenopausal women. Individuals were randomly assigned to combined the herbal capsule and placebo groups. The herbal capsule included cloves (4 units), Anise and Anison (6 units each), violet flowers (12 units), Terminalia Chebula and fresh green raisins (25 units each), senna leaves, Alhagi maurorum, and Golqand (50 units each). Constipation questionnaire and the individual’s assessment of constipation symptoms scale were used for data collection.

**Results:**

The mean frequency of bowel movements in the herbal capsule group was significantly higher than the placebo group (mean difference=4.2; 95% confidence interval: 0.3 to 4.5; p<0.001). Straining during defecation, stool amount, incomplete defecation, sensation of obstruction during defecation, and need for manipulation to facilitate removal of stools were significantly reduced in the herbal capsule group compared to the placebo group (p<0.001). The amount and consistency of defecation was also improved in the two study groups, but a significant difference was observed between the groups (p<0.001). The mean score of constipation symptoms in the herbal capsule group was significantly reduced compared to the placebo group (-15.4; -11.5 to -19.29; p<0.001).

**Conclusion:**

Consumption of herbal capsules improved chronic constipation in postmenopausal women.

## Introduction

Chronic constipation is a common condition with various causes, characterized by poor excretion, low bowel movement frequency, and severe stool congestion (Quigley et al., 2009). Since only a limited number of constipated patients are seeking health care, it is difficult to determine the precise outbreak. The reported incidence rate worldwide is about 13% in children and 33.5% in adults (Mugie et al., 2011). Chronic constipation is a disorder that significantly influences the quality of life of the individual by affecting both physical and mental health dimensions (Belsey et al., 2010). It also reduces individual productivity (Neri et al., 2012) and increases health care costs (Peery et al., 2012).

The criteria for diagnosing chronic constipation according to the Rome III criteria are: bowel movements less than three times a week, straining bowel movements in a minimum of 25% of the time, hard stools with straining in 25% of the time, incomplete excretion in 25% of the time, obstruction of the pathway for excretion in 25% of cases, requiring manipulation to facilitate stool exit in 25% of cases where at least two of the criteria are required during a six month period (Longstreth et al., 2006).

The prevalence of constipation among postmenopausal women has been reported in about 37.3% in two studies (Oliveira et al., 2005; Versi et al., 2001). The incidence of constipation increases with age and various epidemiological studies have found a higher prevalence of constipation in women compared to men (Higgins and Johanson, 2004; Johanson and Kralstein, 2007; Johanson et al., 1989). Even though the association between chronic constipation and sex hormones is not clear, it has been reported that the reduction of ovarian and adrenal steroid hormones is associated with chronic constipation (Tack et al., 2011).

The complications of chronic constipation caused by strain include hemorrhoids, fissures, and pelvic prolapse (Gallagher and Q’Mahony, 2009; Talley et al., 2009). Also, excessive straining in the elderly may also cause a syncopal episode, or coronary ischemia (Gallagher and O'Mahony, 2009). A prospective cohort study on 93676 postmenopausal women has reported that severe constipation is related to enhanced risk of cardiovascular events (Salmoirago-Blotcher et al., 2011). The risk of colorectal cancer also increases due to chronic constipation (Talley et al., 2009).

Common treatments for chronic constipation include increased fluid intake (Gallagher and O’Mahony, 2009), increased fiber intake and the use of laxatives, which include the following types of laxatives: 1) volatile laxatives (bran and methyl cellulose; 2) osmotic laxatives (polyethylene glycol, lactulose, sorbitol); 3) saline laxatives (citrate hydroxide and magnesium sulfate and sodium phosphate); 4) stimulant laxatives (bisacodyl and sodium picosulfate); 5) stool plasticizers (Tack et al., 2011).

There is insufficient evidence supporting the use of large amounts of fluids in the treatment of chronic constipation, and it may cause serious problems in people with renal insufficiency (Gallagher and O'Mahony, 2009; Arnaud, 2003). The use of laxative drugs may also cause unpleasant side effects including nausea and vomiting, diarrhea, abdominal cramps, abdominal distension, and bloating. In addition, salt laxatives cause electrolyte imbalance and long-term safety of laxatives remains relatively unknown (Tack et al., 2011; Ford and Suares, 2011; Leung et al., 2011). In case of volatile laxatives, if the patient is not properly monitored, a possibility of obstruction of the intestine may occur (Salmoirago-Blotcher et al., 2011).

A large number of medicinal plants in traditional medicine sources have been mentioned as having laxative properties. In the book of Qarabadineh Kabir and Mizane Teb, the herbal combination used in this study has been named as a remedy for long-term constipation. The contents of this herbal combination include clover plants, Roman anis or anisone, green anis or fennel, green raisins, Alhagi maurorum, violets, Terminalia chebula, and senna, which were blended with gilt and were used as a capsule (Nazem, 2010).

Clove with the scientific name syzygium aromaticum, has been described in the medical literature as an enhancement of intestinal movements and could improve the digestive capacity of the body by increasing digestive enzymes (Emami et al., 2013; Mozaffarian, 2012; World Health Organization, 1999). Roman anis or anisone (pimpinella anisum) and green anis (foeniculum vulgare) have been introduced in laxative compositions used in traditional medicine in other countries and it has been described as having the ability to increase digestive tract movements (Emami et al., 2013; Mozaffarian, 2012; World Health Organization, 2001). Green raisin, with the scientific name of Vitis vinifera, which contains soluble and insoluble fiber and antioxidants, is presented as a gentle laxative in some sources as well (Mozaffarian, 2012; Ghrairi et al., 2013).

Different types of violet varieties, referred to as viola sp, may stimulate cholinergic activity and reduce constipation (Emami et al., 2013; Mozaffarian, 2012; Muhammad et al., 2013) and terminalia chebula helps to improve constipation by increasing digestive motility (Mozaffarian, 2012; World Health Organization, 2005). Taranbian with the scientific name Alhagi maurorum and golqand have been described as mild laxatives that act through the osmotic process and aid the penetration of water and electrolytes into the intestine (Mozaffarian, 2012; Arezoomandan et al., 2011; Muhammad et al., 2015). The main ingredient of golghand is the petals (rosa damascena), which is combined with honey (Nazem, 2010). In a study, it has been observed that honey also has laxative effects (Ladas et al., 1995). The senna plant is also present in this capsule, which is approximately 20% of the total composition. Considering the reported complications of the celiac herbal remedy, in which the only compound is senna (Santos-Jasso et al., 2017), an herbal mixture based on traditional medicine that reduces the percentage of senna and instead uses gentle laxative herbs as an alternative, seems reasonable.

Therefore, due to the increasing use of traditional medicine and herbal medicines in the treatment of diseases and due to the prevalence of constipation in postmenopausal women and its complications, this research team decided to investigate the effect of the abovementioned combined herbal remedy on chronic constipation in postmenopausal women.

## Materials and Methods

**Study type and participants**

The present study was a randomized, triple blinded controlled trial (participants, researchers, and analysts were blinded to the type of intervention received) that was conducted on 64 constipated postmenopausal women who referred to the health centers of Kermanshah city. The primary outcome of this study was constipation and the secondary outcome was sexual function. The results of secondary outcome have been reported in another article (Eliasvandi et al., 2019) 

The inclusion criteria in this study included: constipated women based on the Rome III criteria, postmenopausal women (who have had their last menstruation at least 12 months prior), age between 40 to 60 years, lack of allergy to the herbal compounds used in this study, married and living with a spouse, having reading and writing skills, having a phone number and willingness to participate in the study. Excluding criteria included suffering from constipation-induced metabolic diseases (hypothyroidism, hyperparathyroidism and diabetes), musculoskeletal disorders (multiple sclerosis, stroke, Parkinson's disease), diarrhea, drug use such as opiates, antidepressants, calcium channel blockers, ibuprofen, anticoagulants, diuretics, and iron and calcium supplements. Suffering from gastrointestinal diseases including colorectal cancer, inflammation and stenosis of duct, taking a drug for constipation within a week before the study and hormone therapy.

According to the previous study on women with constipation by Yang et al. (2008), in which the mean number of bowel movements before the intervention was 2.6, the mean number of bowel movements after the intervention was 4.6, and considering the significant level 0.05, power 90%, and standard deviation of 5.3, the sample size was determined as 30 persons per group, which was considered to be 32 in each group considering the drop out size of the final sample.

**Sampling**

Sampling was started after the study was approved by the Ethics Committee of the Tabriz University of Medical Sciences (ethical code: IR.TBZMED.REC.1395.281) and the study was registered in the Iranian Registry Clinical Trials (IRCT code: IRCT2016043010324N32). The sampling process was conducted from February 2017 to July 2017. Out of 23 health centers of the urban community in Kermanshah, four centers of different socioeconomic levels were selected and participants were selected by the convenience sampling method by referring to the four centers. After explaining the objectives of the study, a checklist of inclusion and exclusion criteria was completed and if the participants meet the eligibility criteria, the socio-demographic questionnaire, constipation questionnaire (CQ) (Mirghafourvand et al., 2016), and Patient Assessment Constipation Symptoms (PAC-SYM) (Frank et al., 1999) were completed through interviews with the women. All women signed the informed consent form.

The constipation questionnaire was distributed to the women for the next two weeks. At the end of the second week, they were re-visited. After receiving the former constipation questionnaire, a new constipation questionnaire was delivered for another two weeks. The PAC-SYM questionnaire was completed for the second time by them. Participants were followed for four weeks. At the end of the fourth week after the delivery of the completed constipation questionnaires, the PAC-SYM questionnaire was completed, and the side effects checklist was completed for the participants.

**Randomization and intervention**

The women were randomly assigned to two groups of intervention (combined herbal capsule user) and control (placebo user) through the blocked randomization method. Block sizes for randomization were four and six, and allocation ratio was 1:1. The herbal and placebo capsules were placed into the closed opaque envelopes numbered sequentially for allocation concealment. Randomization was performed by a person not involved in data collection and analysis. Two small envelopes, each containing a two week supply of the herbal drug or placebo were provided to each participant. The small envelopes were placed into the large opaque envelopes and numbered sequentially. Pockets were opened according to the entry of the individuals into the study, and the researcher delivered a small envelope containing the medication or placebo to the participants sufficient for two weeks. All participants received training on constipation through a pamphlet, including advice on having a high-fiber diet and exercise training. Participants were followed up for four weeks from the time they entered the study. At the end of the second week, the participants were visited and after handing in the CQ questionnaire regarding the two weeks previously, and receiving the second small envelope containing the medication or placebo and the CQ questionnaire for the subsequent two weeks, the PAC-SYM questionnaire was completed through an interview for the second time for the participants. At the end of the fourth week, the participants were re-visited and once again, after handing in the CQ questionnaire and receiving a new CQ questionnaire for another two weeks, the PAC-SYM questionnaire was once again filled out through interviewing all the women. 

The capsules containing the drug and the placebo were prepared completely identical in shape. The correctness of the compounds was confirmed by a pharmacognosy professor and a botanist, and then they were powdered by mill with specific ratios (cloves: 4 units, Anise and Anison: each 6 units; violet flowers: 12 units; Terminalia Chebula and fresh green raisins: each 25 units; senna leaves, Alhagi maurorum, and Golqand: each 50 units). Alhagi maurorum was milled and mixed with Golqand. Then, it took a few hours to spread the Alhagi maurorum's moisture to the Golgand. Next, Golghand was milled with the raisins. Other components were also powdered and mixed with golqand and raisins. Then, it was examined for microbial contamination in a microbial control laboratory to eliminate the microbes, after placing the prepared herbal powder in an oven at 50°C and starch powder in an oven at a temperature of 80°C, an aerobic microbial number count (TAMC) test was performed for both plant and starch specimens. In this test, the number of microbes was less than (Colony-Forming Units per gram) CFU/1000 g.

Escherichia coli and Salmonella bacteria was not present in any of the tested specimens. According to the guidelines in United States Pharmacopeia, the combined herbal powder and starch were both at acceptable levels for microbial control. Moreover, standardization of capsules was carried out in terms of the presence of active ingredient based on the marker compounds in the plants used. 500 mg capsules containing powder of the drug contents were prepared. For the preparation of placebo, 500 mg capsules containing starch were used. The administration of the drug and placebo was done twice a day (before lunch and before dinner) every two capsules for four weeks. Participants were contacted once a week to ensure the drugs were consumed.

**Data collection tools**

Eligibility criteria checklist included a table to assess the eligibility of individuals to participate in the study.

Demographic-social questionnaire: this questionnaire included questions about age, occupation, the adequacy of monthly income for living expenses, the level of education of spouse, housing status, weight, and height. To determine the validity of this questionnaire, it was sent to 10 professors at Tabriz University of Medical Sciences and their comments were applied.

Constipation questionnaire (CQ): included a checklist based on "Rome criteria 3" that was answered by participants for evaluation of constipation before and 1 and 2 weeks after the intervention. This checklist included 7 phrases including the number of stools, the condition of excretion, the stroke of the stool, the incomplete excretion during discharge, the stomach obstruction and the need for manipulation to facilitate the removal of stool. If there were two of the criteria mentioned, the person was suffering from constipation. The validity of this questionnaire had already been confirmed in another study in Iran (Mirghafourvand et al., 2016). 

Patient assessment constipation questionnaire (PAC-SYM): this questionnaire used the 5-point Likert scale, which showed the constipation symptoms severity in the last two weeks in three areas of abdominal symptoms: (four phrases), anal symptoms (three phrases) and feces symptoms (five phrases). The questionnaire was answered before, two weeks, and four weeks post intervention by the women. The validity of this questionnaire had already been confirmed by Frank et al., (1999). In this study, the validity of the translation of this questionnaire was measured through the forward and backward translation method. To assess validity, content and face validity were also used. The content validity reality (CVR) ratio was 0.87 and the content validity index (CVI) was 0.91. A checklist including items about urticaria, itching, abdominal pain, and anus bleeding was also used to record side events. Also, this checklist had an open question for other side effects experienced by participants.

**Statistical analysis**

SPSS software version 21 was used for data analysis. The normality of quantitative data was evaluated by the Kolmogrov-Smirnov test. To compare the consistency of the two groups, the independent t, chi-square, chi-square for trend, and Fisher exact tests were used. The independent t test was applied to compare the number of excretions and the score of constipation symptoms before the intervention, and after the intervention, and the repeated measure ANOVA test was used to control the baseline values. In order to compare the prevalence of straining during excretion, the amount of feces, stool consistency, incomplete emptying, obstruction, and the need for manipulation during excretion, the Mann Whitney U test was used between the groups and the Friedman test was used in the groups. In all the stages, α<0.05 was considered as significant. All analyzes done was based on intention-to-treat.

## Results

**Figure 1:**
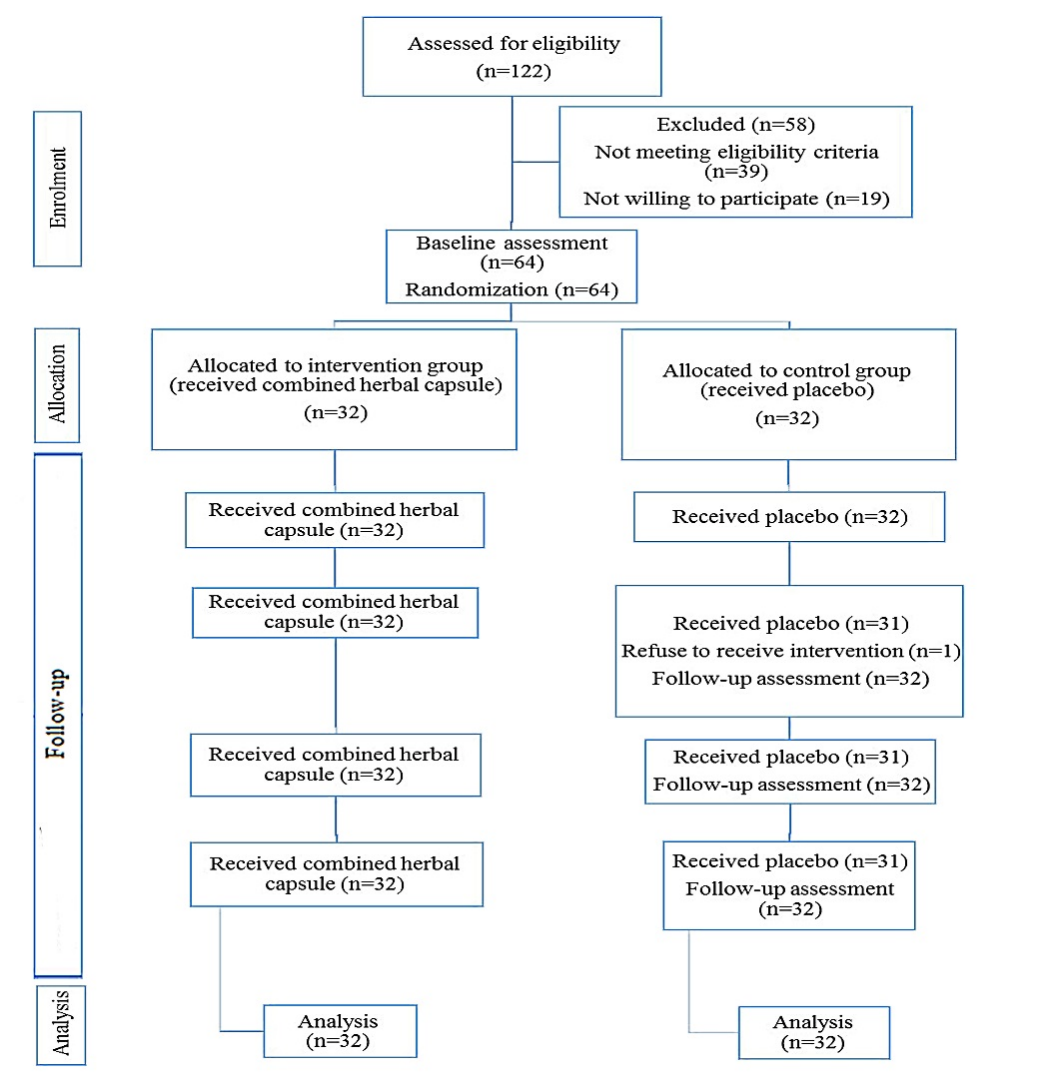
Flowchart of the study

During this period, 122 women were surveyed in terms of inclusion criteria. Of these, 39 were excluded due to lake of eligibility criteria and 19 were excluded due to unwillingness to take part in the study. Sixty-four women were enrolled in the study, 32 patients were assigned to the group receiving the combined herbal capsules and 32 women were assigned to the placebo group and received the intervention. All patients received medications regularly and on time. Only at the end of the first week, one person in the group receiving the placebo refused to continue the intervention, but her follow-up continued until the end of the fourth week. Finally, statistical analysis was performed on data from 32 participants in the herbal capsule group and 32 women in the placebo group (Figure 1). The socio-demographic data of the participants are presented in Table 1. 

**Table 1:** Socio-demographic characteristics of participants by study group

**Characteristics**	**Placebo** **n=32**	**Combined herbal capsule** **n=32**	**p-value**
Age (years)*	52.3 (4.5)	50.5 (4.8)	0.815^†^
Occupation			1.00^‡^
Housekeeper	28 (87.5)	27 (84.4)	
Employee	4 (12.5)	5 (15.6)	
Economic status			0.341^§^
Adequate	17 (53.1)	14 (43.8)	
Relatively adequate	12 (37.5)	17 (53.1)	
Inadequate	3 (9.4)	1 (3.1)	
Education			0.926^§^
Primary school	16 (50.0)	18 (56.3)	
Secondary school	5 (15.6)	4 (12.5)	
High School	6 (18.8)	5 (15.6)	
Diploma	3 (9.4)	1 (3.1)	
University	2 (3.6)	4 (12.5)	
Husband’s education			0.836^§^
Primary school	10 (31.3)	11 (34.4)	
Secondary school	6 (18.8)	5 (15.6)	
High School	7 (21.9)	4 (12.5)	
Diploma	5 (15.6)	6 (18.8)	
University	4 (12.5)	6 (18.8)	
BMI (kg/m2)*	27.8 (3.6)	27.7 (4.2)	0.329^†^
Gravidity			0.409^‡^
≤2	8 (25.0)	9 (28.1)	
3	9 (28.1)	13 (40.6)	
≥4	15 (46.9)	10 (31.3)	
Parity			0.162^§^
≤2	8 (25.0)	11 (34.4)	
3	10 (31.3)	13 (40.6)	
≥4	14 (43.8)	8 (25.0)	
Number of children			0.126^§^
≤2	8 (25.0)	12 (37.5)	
3	10 (31.3)	12 (37.5)	
≥4	14 (43.8)	8 (25.0)	
Sense of health			0.101^§^
Very bad	0 (0.0)	2 (6.3)	
Bad	11 (34.4)	7 (21.9)	
Moderate	19 (59.4)	4 (48.4)	
Good	2 (6.3)	9 (28.1)	
Very good	0 (0.0)	2 (6.3)	

The data indicate number (percent) unless specified as ‘*’

* Mean (standard deviation), ^†^ Independent T-test, ^‡ ^Fisher’s exact test, ^§^ Chi-square test.

The two groups did not have a significant difference in terms of socio-demographic data (p>0.05). The mean (SD=Standard Deviation) of women age in the herbal capsule group was 50.5 (4.2) and 52.3 (4.5) years old in the intervention group. Most of the participants (84.4% in the herbal capsule group and 87.5% in the placebo group) were housewives. More than half of the women (56.3% in the herbal capsule group and 53.1% in the placebo group) had primary education. The majority of participants (96.9% of the herbal capsule group and 90.6% of the placebo group) reported their economic situation as favorable and relatively favorable. The average (SD) BMI in the herbal capsule group was 27.7 (4.2) kg/m^2^ and in the control group was 27.8 (3.6) kg/m^2^. Most of the participants had three birth experiences (40.6% in the herbal capsule group, 31.3% in the placebo group) and more than three births (25.2% in the herbal capsule group and 43.8% in the placebo group). About half of the participants (48.4% in the herbal capsule group and 59.4% in the placebo group) stated their health status as average.

**Figure 2:**
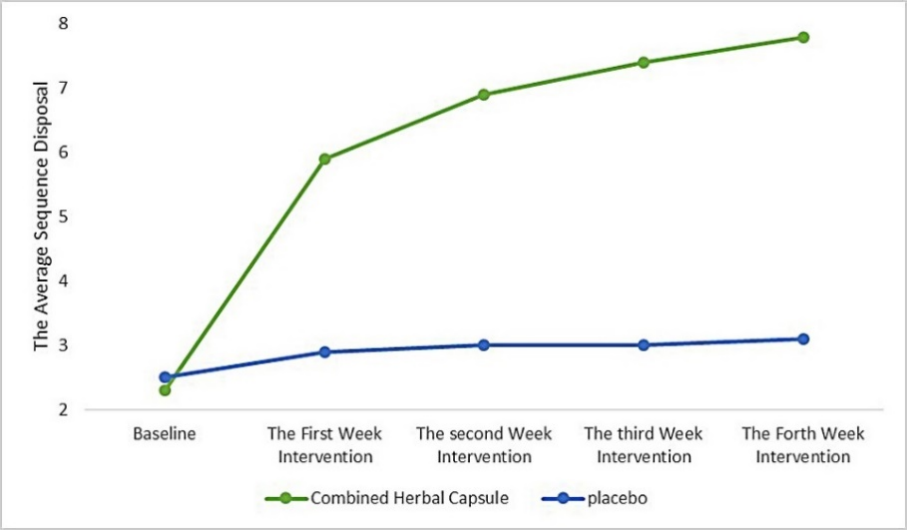
Trend in the frequency of defecation before and during intervention according to repeated measurement analysis

The frequency of bowel movements in the two groups of combined herbal capsules and placebo at the beginning of intervention was not significant. The mean (standard deviation) of bowel movements before intervention in the combined herbal capsule group was 2.5 (0.8) and in the placebo group was 3.2 (0.8). At the end of the fourth week post intervention, the mean (SD) frequency of bowel movements was significantly increased in the combined herbal capsule group, which was 7.8 (3.2) in the combined herbal capsule group and 3.1 (1.8) in the placebo group (p<0.001) (Figure 2, Table 2). There was no significant difference in the prevalence of constipation symptoms between groups before the intervention (p>0.05), but after the intervention, all constipation symptoms included the frequency of straining during excretion, the frequency of low stool, the frequency of tightening stomach consistency, frequency of incomplete excretion, frequency of blockage and abnormalities need to be manipulated during excretion in the first, second, third, and fourth weeks after the intervention, except for the low frequency of stool in the first week after the intervention in the combined herbal capsule group was significantly decreased in comparison to the placebo group (p<0.05). In intragroup comparison, based on the Friedman test, all symptoms of constipation in the combined capsule group indicated a significant improvement (p<0.05) (Table 3).

**Table 2:** Frequency of defecation during intervention in study groups

**Variable**	**Combined herbal capsule group (n=32)** **Mean (SD** * **)**	**Placebo group ** **(n=32)** **Mean (SD** * **)**	**p-value** ^†^	**Time effect (P)** ^ ‡^	**Time and group effect (P)** ^ ‡^
Frequency of defecation in baseline	2.3 (0.8)	2.5 (0.8)	0.283	0.168	0.002
Frequency of defecation in week 1	5.9 (3.2)	2.9 (1.1)			
Frequency of defecation in week 2	6.9 (3.5)	3 (1.5)			
Frequency of defecation in week 3	7.4 (3.7)	3 (1.5)			
Frequency of defecation in week 4	7.8 (3.2)	3.1 (1.8)			
Comparison between groups	MD (CI 95%)^§^ = 4.2 (3.0 to 5.4); p<0.001				

* Standard deviation

^†^ Independent sample T test

^‡ ^Repeated measure ANOVA

^§^Mean difference (Confidence interval 95%)

**Figure 3:**
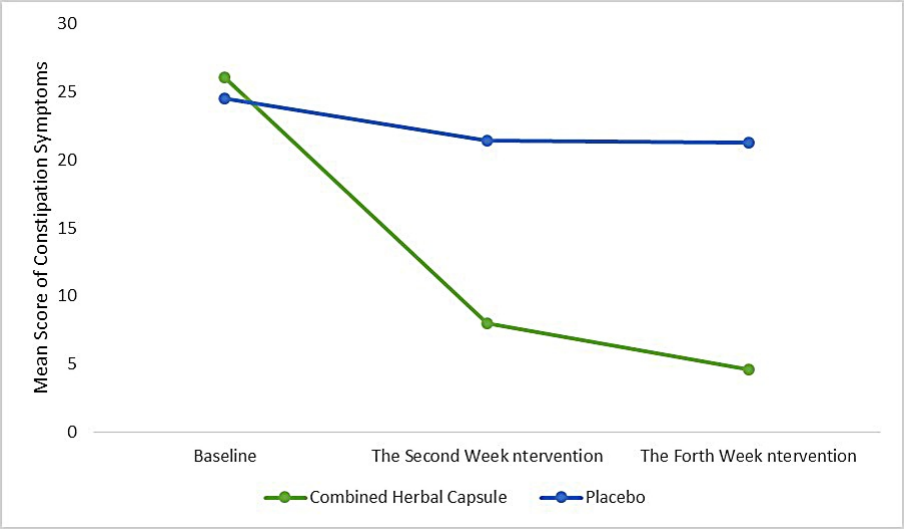
Trend in mean score of constipation symptoms before and during intervention according to repeated measurement analysis

The mean score of constipation symptoms was not statistically significant at the onset of the intervention in the combined herbal capsule and the placebo group. The mean (standard deviation) score of the constipation symptoms before the intervention was 26.1 (8.0) in the combined herbal capsule group and was 25.5 (6.6) in the placebo group and at the end of the fourth week post intervention, the mean (SD) of the symptoms of constipation in the combined herbal capsule group was 4.6 (3.2) and in the placebo group was 21.3 (8.1) which based on the test of repeated measurement ANOVA and control of baseline scores, of the mean score of constipation symptoms in the combined capsule group was significantly lower than the placebo group (adjusted meandifference: -15.4; 95% confidence interval: -19.2 to -11.5; p<0.001) (Figure 3, Table 4).

 Concerning side effects, frequency (percent) of abdominal pain in the placebo group was 2 (6.3) and in the intervention group was 6 (18.8). Anus bleeding was observed in only one person in the placebo group.

**Table 3:** Comparison of constipation criteria between study groups

**Variable**		**Combined herbal capsule group (n=32)** **Number (Percent)**	**Placebo group (n=32)** **Number (Percent)**	**Comparison between groups (p-Value)** ^Ɨ^
The amount of stool in baseline	Few	23 (71.9)	19 (59.4)	0.296
The amount of stool in week 1	Few	5 (15.6)	12 (37.5)	0.36
The amount of stool in week 2	Few	4 (12.5)	13 (40.6)	0.002
The amount of stool in week 3	Few	4 (12.5)	15 (46.9)	< 0.001
The amount of stool in week 4	Few	4 (12.5)	13 (40.6)	< 0.001
Comparison between groups^ǂ^		< 0.001	0.056	
Stool consistency in baseline	Hard	19 (59.4)	26 (81.3)	0.057
Stool consistency in week 1	Hard	12 (37.5)	22 (68.8)	0.003
Stool consistency in week 2	Hard	7 (21.9)	20 (62.5)	< 0.001
Stool consistency in week 3	Hard	5 (15.6)	22 (68.8)	< 0.001
Stool consistency in week 4	Hard	6 (18.8)	21 (65.6)	< 0.001
Comparison between groups^ǂ^		< 0.001	0.012	
Straining during defecation in baseline	Often	11 (34.4)	18 (56.3)	0.103
Straining during defecation in week 1	Often	6 (18.8)	14 (43.8)	0.003
Straining during defecation in week 2	Often	4 (12.5)	17 (53.1)	< 0.001
Straining during defecation in week 3	Often	3 (9.4)	15 (46.9)	< 0.001
Straining during defecation in week 4	Often	2 (6.3)	14 (43.8)	< 0.001
Comparison between groups^ǂ^		< 0.001	0.008	
Sensation of incomplete evacuation after defecation in baseline	Often	9 (28.1)	11 (34.4)	0.053
Sensation of incomplete evacuation after defecation in week 1	Often	3 (9.4)	12 (37.5)	0.008
Sensation of incomplete evacuation after defecation in week 2	Often	8 (25.0)	17 (53.1)	0.001
Sensation of incomplete evacuation after defecation in week 3	Often	3 (9.4)	10 (31.3)	< 0.001
Sensation of incomplete evacuation after defecation in week 4	Often	2 (6.3)	10 (31.3)	< 0.001
Comparison between groups^ǂ^		< 0.001	0.188	
Sensation of obstruction during defecation in baseline	Often	9 (28.1)	11 (34.4)	0.530
Sensation of obstruction during defecation in week 1	Often	3 (9.4)	10 (31.3)	0.004
Sensation of obstruction during defecation in week 2	Often	8 (25.0)	9 (28.1)	0.001
Sensation of obstruction during defecation in week 3	Often	3 (9.4)	12 (37.5)	< 0.001
Sensation of obstruction during defecation in week 4	Often	2 (6.3)	11 (34.4)	< 0.001
Comparison between groups^ǂ^		< 0.001	0.001	
Manual manoeuvers to facilitate defecation in baseline	Often	11 (34.4)	4 (12.5)	0.422
Manual manoeuvers to facilitate defecation in week 1	Often	4 (12.5)	8 (25.0)	0.001
Manual manoeuvers to facilitate defecation in week 2	Often	3 (9.6)	4 (12.5)	< 0.001
Manual manoeuvers to facilitate defecation in week 3	Often	2 (6.3)	4 (12.5)	< 0.001
Manual manoeuvers to facilitate defecation in week 4	Often	3 (9.4)	4 (12.5)	< 0.001
Comparison between groups^ǂ^		< 0.001	0.006	

Ɨ Measured by Mann-Whitney U

ǂ Measured by Friedman

**Table 4:** Mean score of constipation symptoms during intervention in study groups

**Mean score of constipation symptoms**	**Combined herbal capsule group (n=32)** **Mean (SD** * **)**	**Placebo group ** **(n=32)** **Mean (SD** * **)**	**p-value** ^†^	**Time effect (P)** ^ ‡^	**Time and group effect (P)** ^ ‡^
in Baseline	26.1 (8.0)	24.5 (6.6)	0.362	0.744	<0.001
Week 2	8 (8.0)	21.4 (7.4)			
Week 4	4.6 (3.2)	21.3 (8.1)			
Comparison between groups	MD (CI 95%)^§^ = -15.4, (-19.2 to -11.5); p<0.001				

* Standard deviation

^†^ Independent sample T test

^‡ ^Repeated measure ANOVA

^§^Mean difference (Confidence interval 95%)

## Discussion

In this study, the combined herbal capsule significantly improved constipation and associated symptoms based on the Rome III criteria compared to the placebo.

 This is the first time that this herbal compound capsule has been scientifically studied; however, the results of other studies which used the herbs in this compound separately have been evaluated and its effect on chronic constipation has been reported.

In a similar research done by Picon et al. (2010) in Brazil on 20 patients with chronic constipation, the effect of herbal tea composed of Foeniculum vulgare, Sambucus nigra and Cassia agustifolia was compared with a placebo. Half of the patients were given the herbal tea for five days and the other half received the placebo. After that, both groups spent nine days washing out, and in the next step, the intervention and control groups were changed. This time the first group received the placebo and second group received the herbal compound. The main goal was to measure colon transfer time (CTT) by radiology. Secondary goals included the frequency of bowel movements per day, the percentage of abdominal function, side effects, and quality of life. The average time for colon transection was 7.7 hours in the herbal compound group and 3.42 hours in the placebo group. The frequency of bowel movements per day was increased during the use of herbal teas (p<0.001). Improvement of intestinal function of patients during the treatment period was significantly different in the herbal compound group compared to the placebo group (p<0.001). However, the quality of life did not indicate significant statistical changes during the period. In another placebo-controlled randomized clinical trial by Bub et al. (2006), 86 nursing home residents with chronic constipation were randomly allocated to the Smooth Move herbal tea group or placebo tea group. They received the drug or placebo once daily for 28 days. Each single serving of herbal tea contained 1080 mg of the stimulant laxative active ingredient senna leaf PhEur (Cassia angustifolia Vahl). The results showed that Smooth Move herbal tea increased the mean number of bowel movements compared to the placebo tea. The findings of the two above mentioned studies indicate that taking an herbal tea was effective on improving chronic constipation, which is consistent with the findings of the present study.

The study of Kazerani et al. (2009) in Iran assessed the laxative and purgative effects of Alhagi maurorum (Taranjabin) on rats. The single dose of 2.5 g/kg of Taranjabin was gavaged to seven healthy rats to evaluate its laxative and purgative effects according to the feces count and weight and its water percent during the next 24 hours after treatment. The seven rats in the control group received a placebo by the same way. The feces count was more in the treatment group during the second 8-hour after the treatment. The feces weight and its water percentage was significantly higher in the treatment group during the second and third 8-hour compared to the placebo group. These findings show a laxative effect for the Alhagi in rats, which is consistent with the results of our study.

In another study was conducted by Muhammad et al. (2013) in Pakistan to study the effect of violet extract on mice. The violet extract is rich in alkaloids and saponins, and with its cholinergic effects led to the production of moisturized feces and increased intestinal movements in mice. This study recommended taking violet as a drug to reduce indigestion and constipation.

The study of Arzoumand et al. (2010) in Iran was conducted to investigate the prokinetic and laxative effects of Rosa damascene on mice. In this study, 14 mice were entered in the study; half of them received a Rosa damascena extract and the other half received a placebo by gavage. Then the number, weight, and percentage of stool water were evaluated for up to 24 hours. The results showed that Rosa damascena extract increased the frequency of bowel movements, moisture and stool volume compare to the placebo group. The proposed mechanism of this study was to stimulate the osmotic penetration of water into the intestine, which is consistent with the mechanism and results obtained in our study. 

A study by Ladas et al. was done on 20 healthy people (7 women and 13 men) without any illness in Greece regarding the effect of honey on intestinal symptoms and carbohydrate metabolism. In this research, it was observed that the participants who consumed honey were more likely to suffer from diarrhea, which could be due to the laxative effects of honey (Ladas et al., 1995). In the study, honey probably helped to reduce the symptoms of constipation through the same mechanism. Unfortunately, valid studies which measure the effects of some of the herbal products in this compound have not been found to be compared with the results of this study.

One of the limitations of this study is that, considering that the participants were selected among postmenopausal women in governmental health centers, this could affect the generalizability of the results. However, to address this problem, sample the centers with the different social and economic levels were selected. Another limitation is that hemorrhoid was not assessed in this study to exclude the women who had this problem. One of the strengths of the study is its triple-blindness and following all principles of the randomized controlled trial including randomization and allocation concealment. It is recommended to evaluate the effect of this capsule on chronic constipation of people of different ages, as well as its long-term effects.

In this study, the use of herbal capsules resulted in the improvement of constipation and its symptoms. Considering the high prevalence of constipation, the side effects of laxative drugs, and the lack of observation of side effects in the combined herbal capsule used in this study, it is recommended to use this capsule to treat constipation.
